# A Splenic Abscess in a Trauma Setting: A Case Report and Comprehensive Literature Review

**DOI:** 10.7759/cureus.54866

**Published:** 2024-02-25

**Authors:** Salam Tooza, Nicholas Lee

**Affiliations:** 1 General Surgery, John Hunter Hospital, Newcastle, AUS; 2 Trauma Surgery, John Hunter Hospital, Newcastle, AUS

**Keywords:** management of traumatic splenic abscess, total splenectomy, percutaneous abscess drainage, traumatic splenic abscess, management of splenic abscess

## Abstract

A splenic abscess is rare after trauma, and it has been reported with high mortality rates. Treatment options include antibiotics, percutaneous pigtail drain, or surgical intervention; however, there are no specific guidelines for the treatment of splenic abscesses in trauma settings. We report a 69-year-old male who came in with a splenic abscess after angioembolisation for a splenic laceration after having a right hemicolectomy. Our case presents new information and current recommendations for the management of splenic abscesses based on a comprehensive literature review.

## Introduction

Splenic abscesses are rare worldwide, with an incidence of 0.05-0.7% [1]. Despite antibiotics, splenic abscesses still have a high mortality rate (41-100) [2]. Hagler et al. report that factors contributing to splenic abscesses include trauma, iatrogenic injury, sickle-cell disease, embolic infection, immune compromise, and immunodeficiency. Increased use of corticosteroids, chemotherapy, immunomodulators, organ transplantation, and acquired immunodeficiency syndrome (AIDS) or hematological disorders increase the risk [3-5].

VanSonnenberg et al. found that intraabdominal abscesses can be treated in various ways, with a single mature abscess cured in up to 80-90% of patients [6]. Patients who have undergone percutaneous drainage may avoid surgery [6]. Treatment options include broad-spectrum antibiotics and percutaneous drainage [7]. In one-third of cases, splenectomy and laparotomy are necessary, especially in trauma settings [6]. Risk factors for failure and complications include age, injury grade, presence of a pseudoaneurysm, and traumatic injuries.

Percutaneous drains can serve as a bridge to surgery for critically ill patients with multiple comorbidities [8], but they carry risks such as bleeding, organ damage [9], and colonic damage [10], which must be carefully considered.

## Case presentation

A 69-year-old man with a history of deep vein thrombosis, peripheral vascular disease, and hypertension was evaluated in the emergency room four months after a right-sided hemicolectomy for invasive, low-grade ascending colon adenocarcinoma. He presented with upper abdominal pain and vomiting. Radiological tests revealed a large splenic hemorrhage with a large volume of hemoperitoneum. He also had a small bowel obstruction with a transition point in the right iliac fossa.

**Figure 1:**
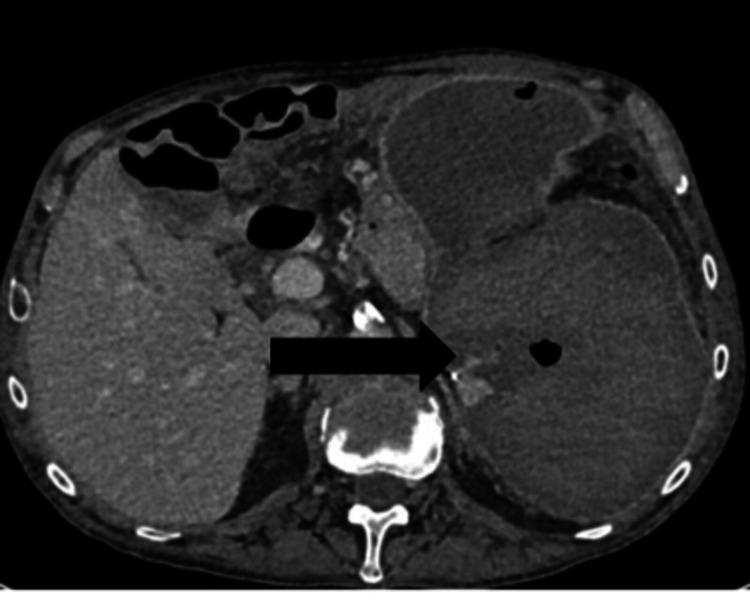
CT abdomen showing gas locules in the large splenic hematoma

Initially, he was treated with distal embolization of the peripheral branch, and the bowel obstruction was resolved and discharged. Three weeks after admission, he presented with left upper quadrant pain, leucocytosis, and fever. A CT scan revealed a large 200-mm walled-off collection/hematoma with scattered gas locules, a very small left basal pleural effusion, and mild atelectasis (Figure 1).

A decision was made for percutaneous drainage insertion, and a 12 Fr Dawson Mueller drainage catheter was inserted over a guidewire. The splenic fluid MSC (Clostridium tertium) was isolated and treated with Augmentin and oral antibiotics. The percutaneous drain was kept for a month, and the drain was removed after full recovery.

## Discussion

The study by Lee et al. found that 4.6% of patients with blunt trauma had intra-abdominal abscesses [11], which are often linked to splenic and liver damage [12].

The mortality rate of splenic abscesses is still high, and early detection is crucial. Fever, left upper quadrant pain, and leucocytosis are present in one-third of the cases [13]. The patient had recent abdominal surgery, trauma, and angioembolization, which are risk factors for splenic abscess. The CT scan revealed a gas locule inside the splenic hematoma, making the diagnosis challenging.

Treatment options vary based on patient stability, comorbidities, and the severity of the injury. Splenectomy is the standard treatment, but antibiotics and percutaneous drainage can be successful [10]. Eight point four percent (8.4%) of patients experienced minor complications such as catheter kinking, displacement, or blockage due to thick fluid, flakes, and debris [14].

According to our review of the literature, there have been 13 cases of splenic abscesses in trauma settings (Table 1), and patients may experience these abscesses 48 hours to 4 months after the trauma. In this case, multiple factors, including recent abdominal surgery and improved splenic abscess drainage, suggest that percutaneous drainage and possibly the insertion of another drain are reasonable approaches to splenic abscesses, even in trauma settings.

**Table 1:** Review of the literature for splenic abscesses after trauma

Author/Citation	Article Title	Age	Comorbidities	Mechanism	Symptoms	Images	Treatment	Days till develop Splenic Abscess	Complication	Year
Sands M et al. [15]	Splenic Abscess Following Nonoperative Management of Splenic Rupture	16	Unremarkable	Blunt trauma to his left chest while playing football.	Left upper quadrant abdominal pain and left shoulder pain exacerbated by coughing.	CT scan was performed, which demonstrated a hypodense area at the inferior splenic pole containing gas.	Splenectomy	15 days	Unremarkable	1984
Nikolaidis et al. [16]	Post-traumatic Splenic Abscess With Gastrosplenic Fistula	70	Non-insulin-dependent diabetes mellitus of 30 years duration and paroxysmal atrial fibrillation.	Fall from a tree of height 1.5 m, landing on his left side.	Left hypochondrial pain, malaise, and weight loss of 15 kg.	CT: a large area within the spleen of low attenuation, with a thin, air-filled, fistulous tract between the gastric lumen and the splenic cavity. The transabdominal US of the spleen revealed no septation.	CT-guided percutaneous drainage was performed; after that, the patient underwent splenectomy together with resection of the fistula and part of the gastric fundus.	2 months	Unremarkable	2005
Hagler et al. [2]	Splenic Abscess Requiring Early Splenectomy Following Angioembolization for Blunt Splenic Injury in an Immunocompromised Host: Implications for Management	66	HIV and type II diabetes mellitus.	Blunt polytrauma after a fall.	Unstable, abdominal distention.	Initial CT Grade III splenic laceration with active extravasation, repeat CT within 48 h splenic necrosis with significant amounts of air within the splenic parenchyma and splenic fossa.	Laparotomy and splenectomy.	48 hours	Unremarkable	2016
Toevs et al. [17]	Splenic Abscess 10 Years After Splenic Trauma: A Case Report	47	Hypertension.	Snowmobile accident more than 10 years ago.	Tingling in her arms and legs, pain under her left breast, and chills.	A CT scan was obtained, which revealed a splenic abscess.	Splenectomy.	10 years	Nothing reported	2000
Ng Liet Hing C et al. [18]	Actinomycosis of the Spleen Following Splenic Artery Embolization in the Setting of Trauma	37	Intravenous drug abuse, hepatitis C.	Motorbike crash at 30 km/h.	Fever and abdominal pain.	Initial CT grade V splenic injury with large volume hemoperitoneum - repeat CT pneumoperitoneum with the majority of the spleen being replaced by a necrotic non-drainable collection.	Selective coiling of the upper pole vessel was carried out with good angiographic results for the bleed, and then splenectomy.	9 days	Nothing reported	2020
Liu S et al. [19]	Splenic rupture, liquefaction, and infection after blunt abdominal trauma.	48	Unremarkable.	Mechanical fall 2 weeks prior to presentation.	Fever and abdominal pain.	CT unenhanced lower pole of the spleen demonstrated liquefaction with rupture through the lateral capsule and hyperdense layering and fluid tracking along the diaphragm, paracolic gutter, and pelvis suspicious for subacute hemorrhage with superimposed infection. Moderate left hydroureteronephrosis without an obstructing lesion consistent with chronic ureteropelvic junction obstruction was also identified.	Splenectomy.	2 weeks	Nothing reported	2018
Moghimi Z et al. [20]	Splenic Abscess Due to Non-operative Management of Splenic Injury: A Case Report	68	Ischemic heart disease, diabetes mellitus, ejection fraction ≤35%, but negative past surgical history.	Blunt abdominal trauma 2 weeks ago.	Generalized abdominal pain and fever of 39 degree centigrade.	Initial CT splenic subcapsular hematoma (grade 3); then a CT scan showed a large splenic abscess measuring 10.8×8.7×5.3 cm (centime).	Splenectomy)	2 weeks	Nothing reported	2023
de Wit CWM et al. [21]	Splenic Abscess Following Non-operative Treatment of a Splenic Rupture Caused by Blunt Abdominal Trauma	35	Non-operative treatment.	Blunt injury of the left abdomen caused by an accident with his motorcycle.	Fever, left upper abdomen, and left shoulder.	CT scan of the abdomen showed a multifragmentation rupture of grade III of the spleen.	CT-guided percutaneous drainage of the abscess followed and a 7 French drain was left in situ.	12 days	NA	1994
Collis J et al. [22]	Splenic Abscess Formation Following Selective Splenic Embolisation for a Traumatic Splenic Injury and Its Subsequent Conservative Management	50	NA	NA	NA	CT grade IV splenic laceration, then a splenic abscess with worsening of his pleural effusion.	Interventional drainage	>7 days	Nothing reported	2022
Kumar A et al. [23]	Splenic Abscess Following Blunt Abdominal Trauma	15	NA	Falling off a bicycle.	High-grade fever and severe pain in the left hypochondrium for 6 days.	Sonography of the abdomen demonstrated a large hypoechoic area on the anteromedial aspect of the spleen and fluid collection in the left subdiaphragmatic space and peritoneum.	Splenectomy	1 week	NA	1995
Milad N et al. [24]	Incidence of Splenic Abscess After Conservative Management of Blunt Splenic Injury: A Cross-Sectional Study	The two cases of splenic abscess developed fever and left hypochondrial pain.					Open splenectomy because the patient had multiple abscesses and the other case by splenectomy after failed ultrasound-guided aspiration because the abscess was multiloculated.		NA	2022
Demma J et al. [25]	Splenic Infarction Complicated With Abscess After Pelvic Trauma As the First Presentation of Patent Foramen Ovale – A Case Report	35	Bipolar disorder and type 2 diabetes mellitus.	Hit by an electric bicycle.	Epigastric pain.	Initial CT showed sustained fractures of the Ramus pubis and transverse process of the fifth lumbar vertebrae as well as a grade 1 tear of the spleen then another CT showed a splenic abscess and a small amount of free intraperitoneal air.	Splenectomy	2 weeks	NA	2021
Sreekar H et al. [26]	A Retrospective Study of 75 Cases of Splenic Abscess					Two cases of abscesses with trauma were documented.	One was treated with a percutaneous drain and one with splenectomy.		NA	

## Conclusions

A splenic abscess in trauma settings is a rare condition that requires prompt diagnosis and intervention. There is no gold standard for treatment, and management options should be tailored to each patient. Percutaneous drainage with one or two drains is a feasible option.
